# Adjacent Implant Influence on Tooth Restoration in a Gastro-Esophageal Reflux Disease (GERD) Patient Using Monoblock Post-Fused Crown: A Case Report

**DOI:** 10.7759/cureus.56482

**Published:** 2024-03-19

**Authors:** Navdeep Jethi, Subhankar Banik, Divya Nagri Bhan, Rachana Mishra

**Affiliations:** 1 Conservative Dentistry and Endodontics, Daswani Dental College and Research Centre, Kota, IND; 2 Conservative Dentistry and Endodontics, Agartala Government Dental College and IGM Hospital, Agartala, IND; 3 Prosthodontics, Institute of Technology & Science (I.T.S) Dental College, Ghaziabad, IND

**Keywords:** galvanism, gastroesophageal reflux disease (gerd), post endodontic restoration, endodontic treatment, dental impression technique, dental occlusion, monoblock concept, factors determining dental implant therapy, dental aesthetics, post and core technique

## Abstract

This case report explores how the presence of an adjacent implant influenced the restoration of a tooth with pulpitis in a gastro-esophageal reflux disease (GERD) patient. A patient with GERD requires a tooth-colored crown not only for aesthetics but also to address potential galvanic concerns arising from an adjacent implant. GERD, a condition causing non-cavity erosion, weakens tooth structure over time, presenting significant challenges in treatment. It resulted in bite relapse and insufficient occlusal clarity over time. A comprehensive treatment approach was needed to restore both function and appearance. This involved managing galvanism using non-metallic materials to ensure optimal occlusal clarity, as well as meticulously reinforcing and restoring tooth structure. Monoblock post-fused crowns were chosen for their superior durability, stability, and comfort. The ceramic layering not only effectively prevented galvanic issues by insulating the tooth structure but also significantly improved the natural appearance of teeth, thereby promoting long-term oral health and successfully managing complex dental concerns. The dental team successfully restored the damaged tooth by considering specific factors that influenced the treatment plan, including achieving optimal aesthetic outcomes.

## Introduction

A good endodontic treatment enhances pain relief, and a good prosthetic replacement maintains occlusion and chewing function [1]. Tooth-colored restorations are aesthetically pleasing but require adequate crown length and occlusal clearance (2 mm) [2] between opposing teeth in the upper and lower arches following crown preparation (occlusal and axial reduction) [1]. Furthermore, non-carious lesions, such as attrition and erosion, may complicate treatment plans for compromised teeth by compromising structural integrity, potentially resulting in bite collapse and deep bites [3].

In situations where there is sufficient occlusal clearance, tooth height can be increased through post-core buildup. However, inadequate clearance may necessitate surgical or laser crown lengthening [1,4]. In some cases, surgical crown lengthening and subgingival margins can lead to potential gingival inflammation [5]. Unaesthetic metal crowns are often recommended for back teeth with inadequate occlusal clearance [1,6]. In cases of severely damaged teeth with minimal height and compromised structure due to decay, the retention of a crown becomes uncertain due to compromised integrity and limited support [4]. Post and core buildups are essential in such scenarios to strengthen the weakened tooth structure and improve crown retention [6]. Therefore, in such cases, post-endodontic restoration with a monoblock post-fused crown is recommended to ensure long-term structural stability and functional restoration, as it offers superior support and durability for the weakened tooth structure. In this process, a tooth’s post and crown are cast together as one piece to address issues with occlusion clearance. They are then covered with tooth-colored ceramics for better aesthetics and seamless blending with natural teeth [6,7]. This is not a traditional Richmond crown [8] or the typical modification used for anterior teeth with ceramic facings [9].

When planning dental treatment, the dentist considers both the patient’s medical history (overall health) and dental history to tailor a personalized treatment approach [10,11]. The absence of a crown on the implant for more than six months caused decay in the adjacent mandibular premolar, leading to pulp infection. As per research by Smith et al. (2020), a tooth adjacent to an implant can decay over time due to changes in jaw structure and the absence of crowns on implants [10].

In GERD, refluxed acid first attacks the palatal surfaces of the upper incisor teeth. If the condition persists, erosion of the occlusal surfaces of the posterior teeth in both arches and the labial or buccal surfaces occurs due to prolonged acid reflux [11,12]. Also, poor oral health due to plaque and calculus accumulation on the lingual surfaces of mandibular teeth and acid corrosion of the metal crowns can be seen in GERD patients [12].

Considering the patient’s history of gastroesophageal acid reflux [11] and existing dental implants, the nearby prosthesis made of “all metals” could potentially lead to galvanism [13]. As highlighted by Kim SM (2023), contact between two different metal alloys in the presence of saliva initiates electrochemical reactions [13]. This phenomenon can result in galvanic erosion, additional soft tissue problems, tooth erosion, crown corrosion, and peri-implantitis [13]. Therefore, in light of the patient’s color preference, concerns regarding GERD, and the risk of galvanism, a monoblock post-fused porcelain crown was selected for this case.

## Case presentation

Patient history

A 42-year-old highly educated woman presented to the Department of Conservative Dentistry and Endodontics, with a chief complaint of pain and food accumulation in the tooth located on the right side of the lower jaw. The pain worsened with the consumption of tea and puddings. In terms of her dental history*, *an implant was placed in the same quadrant to replace the first molar (tooth 46) six months ago in the Prosthodontics Department. However, the patient was unable to receive a crown due to travel obligations. Prior to implant placement six months ago, when she was examined in the conservative dentistry department, she had plaque and calculus in the lingual and palatal aspects of her upper and lower teeth. There were no caries on the lower right second premolar, and there was a missing tooth in the right molar place. She began to notice changes in her lower premolar tooth after she ignored brushing and eating from the implant side during her healing period. Additionally, the patient used abrasive powder for teeth cleaning, which has the potential to erode enamel and irritate gums. She gave no history of grinding teeth at night, and when cross-questioned, his husband said he had never heard a grinding sound by her side of the bed. She took 6-7 cups of tea in a day. She initially consumed acidic beverages like soft drinks and lime juice to manage acidity and gas, but now reduces her intake due to ill effects. She underwent scaling two months ago on dentist recommendations for plaque and calculus over the palatal and lingual areas. In her medical history, the patient had GERD, which caused nocturnal acid reflux while lying down, leading to erosion. She was under the care of a gastroenterologist and prescribed omeprazole 30 mg twice daily to manage heartburn and acidity.

Clinical examination

**Figure 1:**
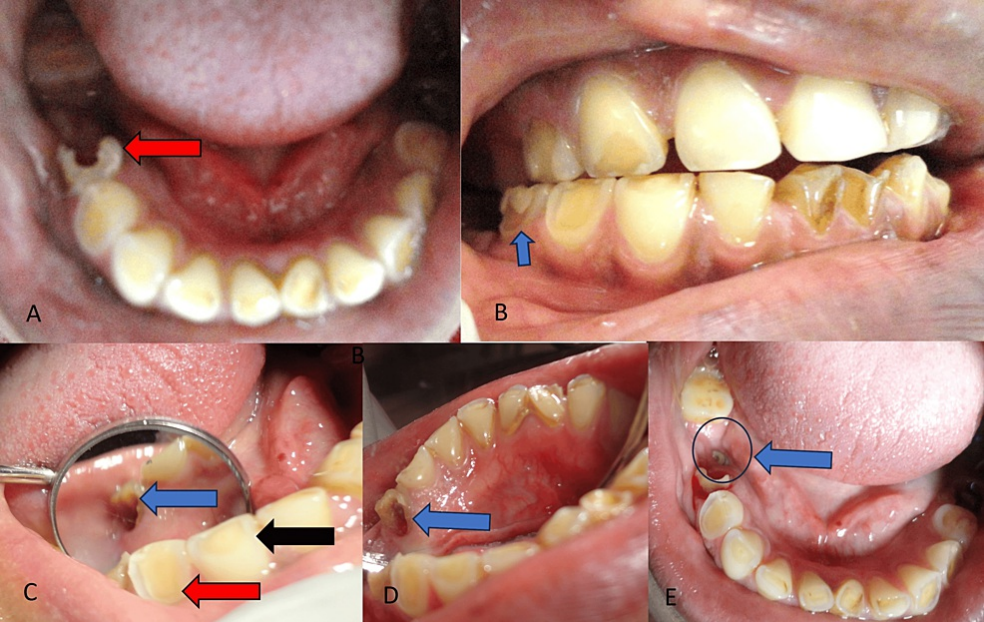
Preoperative photographs A) Disto-proximal caries in tooth 45 (red arrow); B) Posterior bite collapse due to generalized attrition and erosion; erosion and attrition in tooth 45( blue arrow); C) Dental caries in tooth 45 (blue arrow); labial side enamel loss due to erosion in tooth 44 (red arrow); occlusal wear facets in tooth 43 (black arrow); D) Buccal and proximal wall of tooth damaged in tooth 45 (blue arrow); E) Implant without prosthesis in tooth 46 (blue arrow); and generalized enamel loss due to attrition and erosion in mandibular dentition.

During the clinical examination, an intraoral assessment revealed widespread attrition and erosion of the dentition (Figure 1A). Additionally, cup-like deformities were observed on the occlusal surfaces of the mandibular dentition (Figure 1A). Furthermore, the enamel of the central incisors was completely eroded (Figure 1B), and posterior bite collapse was evident (Figure 1B). The painful tooth adjacent to the implant without a crown in the 46 regions, which was placed approximately six months ago, was identified (Figure 1E). A carious lesion, characterized by a moderate-sized cavity, was noted on the distal proximal side of the lower right second premolar (tooth 45) (Figures 1C-1E). This lesion was absent when she was diagnosed six months prior to implant placement. Additionally, gross enamel loss was observed on the labial aspect of these teeth, with a yellowish dentin color visible (Figure 1C-1E). Surprisingly, no lingual or palatal erosion was observed, making this a rare finding for GERD patients [11,12]. Damage was evident on the labial and distal proximal sides of the tooth (Figures 1C-1E). The lower right second premolar tooth exhibited mild tenderness to percussion but remained non-mobile, indicating stable positioning. Pulp vitality was assessed using an electric pulp tester, confirming the presence of a vital pulp.

Intraoral radiographic interpretations

**Figure 2:**
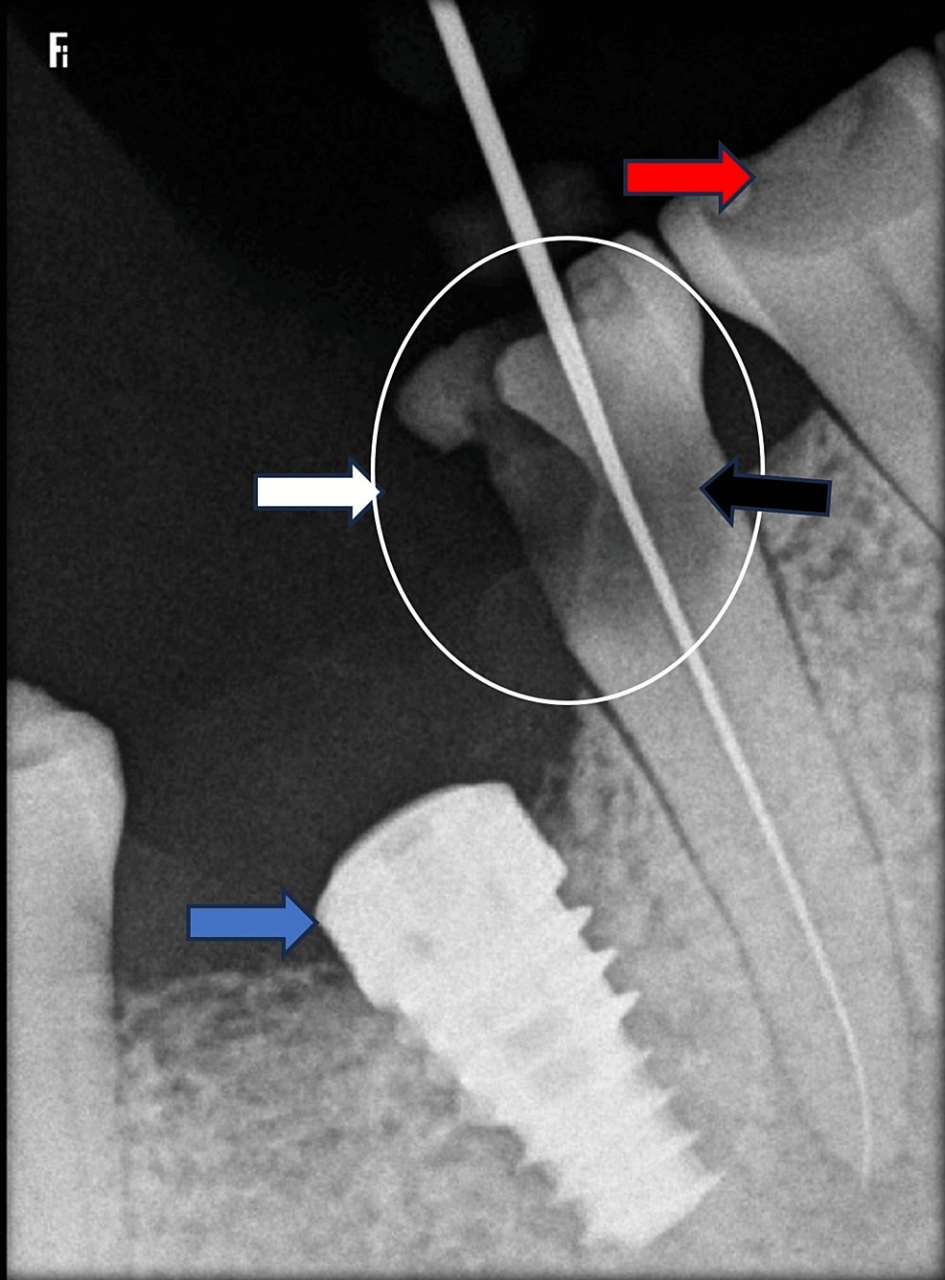
Radiographic interpretations Crownless implant in tooth 46 (blue arrow); Disto-proximal Caries in tooth 45 (white arrow and circle); Erosion in tooth 45 (black arrow); and Erosion in tooth 44 (red arrow).

Disto-proximal caries with radiolucency have approached the pulp in the single-rooted right mandibular second premolar (tooth 45) without periapical involvement. Adjacent to the lower right second premolar, an osseo-integrated implant (tooth 46) (indicated by the blue arrow) was identified (Figure 2). Additionally, radiolucency is evident, encroaching upon the enamel and dentine of tooth 44 due to attrition and erosion, potentially resulting in heightened sensitivity and structural weakening.

Treatment plan

The initial treatment plan for this tooth was to perform endodontic treatment on the lower right second premolar, tooth 45, due to pulp involvement. Subsequently, a crown was planned to restore the tooth following the root canal treatment. Considering the limited vertical space between the jaws, upon the removal of decay, it was discovered that two walls of the tooth were damaged. Consequently, a post and core buildup was deemed necessary to reinforce the remaining tooth structure and provide support for the crown.

During the consultation, the patient expressed a preference for a tooth-colored crown over silver teeth. In response, the dental team scheduled a monoblock post and crown to address concerns regarding poor occlusal clarity and to meet aesthetic requirements. Additionally, the dental specialist advised against using an all-metal crown adjacent to an implant to mitigate potential issues such as non-carious lesions, GERD [14], and galvanic erosion [13].

GERD management

**Figure 3:**
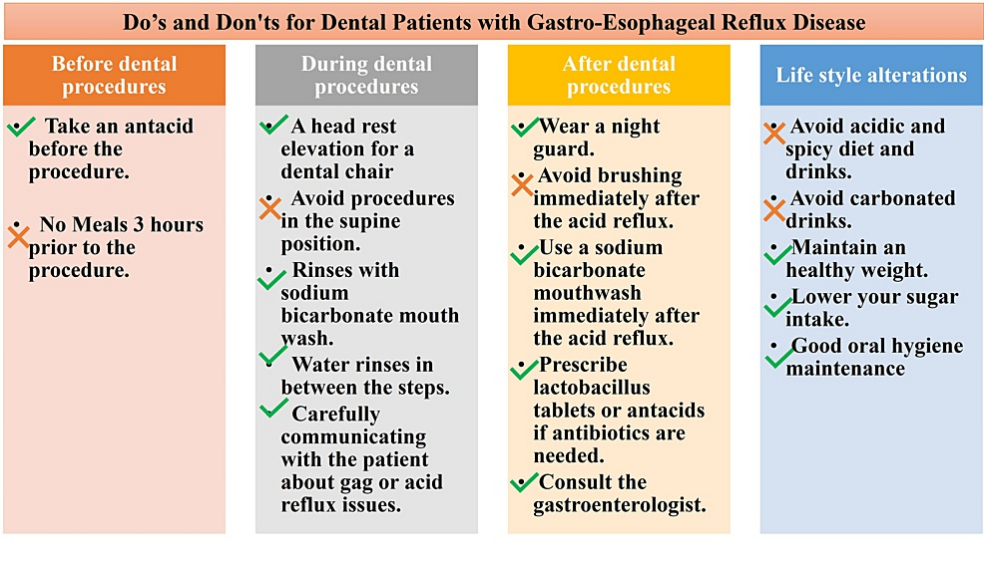
Considerations for dental procedures in gastro-esophageal reflux disease patients This list was initially devised and followed in this case by the author Navdeep Jethi.

As the patient consulted her gastroenterologist for acid reflux, several considerations were followed throughout the procedure (Figure 3).

Endodontic treatment

**Figure 4:**
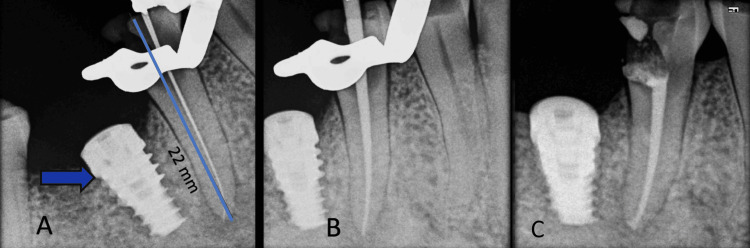
Endodontic treatment images A) Implant in tooth 46 (blue arrow), working length of tooth 45: 22 mm; B) Master cone in tooth 45; C) Obturation in tooth 45.

Oral and written consents were obtained prior to the procedure. Under local anesthesia and rubber dam isolation, caries were meticulously removed from the proximal aspect of the right lower second premolar (tooth 45). Extensive loss of tooth structure was observed post-caries removal (Figure 4A). Access openings were created using round burs (sizes 2 and 4) along with Endo Z burs. Subsequently, remnants of the pulp tissue were carefully extirpated. A glide path was established utilizing 6K, 8K, and 10K files, which were lubricated with ethylene diamine tetraacetic acid (EDTA) gel. Irrigation was carried out using normal saline and a 3% NaOCl solution. The working length was determined with a 15K file within the canal, utilizing an apex locator, and further confirmed at 22 mm (Figure 4A) employing Vatech digital radiography software (EZsensor, Korea). Initial canal enlargement was performed with 15-30K files, aiming for straight access to the apex. Cleaning and shaping were executed using Protaper Gold rotary files in conjunction with Endomotar. The root canal orifice was enlarged using Sx and S1 files, followed by shaping with sizes F1 to F4. Normal saline and a 3% NaOCl solution were alternately used to irrigate the canal, ensuring comprehensive debris removal at each step. Subsequently, the canal was dried using paper points and aspiration with a syringe. The obturation procedure was conducted using F4-size gutta-percha and Ah Plus sealer during the same visit [15], ensuring thorough sealing of the canal to prevent any potential leakage (Figures 4B-4C).

Post-space preparation

**Figure 5:**
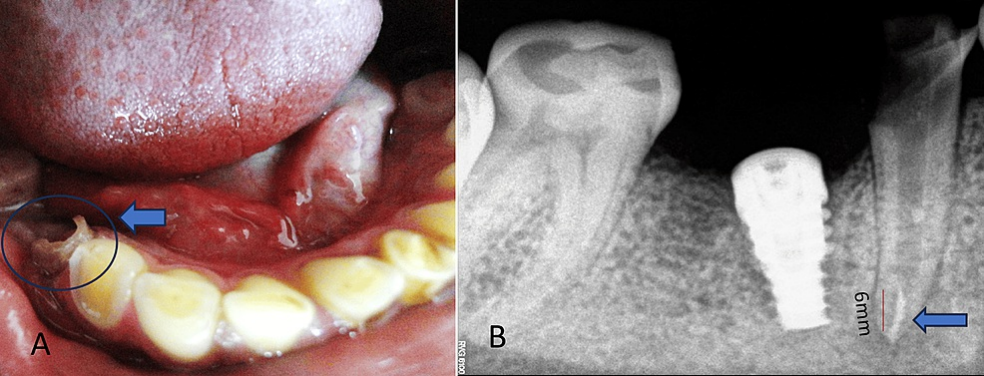
Post-space preparation A) Crown preparation and ferrule preparation for 45 (blue arrow and circle) B) Post-space preparation up to 16 mm and 6 mm gutta percha left for apical seal (blue arrow).

The patient was recalled after seven days, and no pain or tenderness was noted upon percussion. During the second visit, a conservative ferrule was prepared on the tooth with a chamfer margin finish (Figure 5A). Peeso reamers (sizes 1-4) were utilized within the canal to remove gutta-percha for post-space preparation up to 16 mm. As it is recommended to retain 4-6 mm of gutta-percha at the apex for optimal sealing, the apical 6 mm of gutta-percha was left in place within the canal for apical seal (Figure 5B).

Impression making

**Figure 6:**
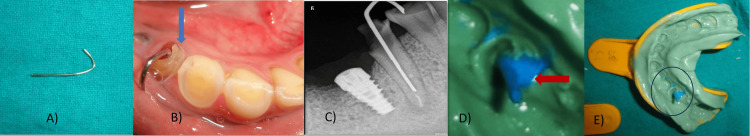
Post space impression procedure to make a custom single unit post fused crown A) J-shaped wire; B) wire placed in tooth 45 to check the length (blue arrow); C) radiograph with J-shaped wire inside the tooth; D) post-space impression made with a light body (red arrow); E) impression made with J-shaped wire in the root canal (blue circle).

A 19-gauge wire was shaped like a J and utilized to create an impression of the space in the canal where the post fits (Figures 6A-6C). The light body was mixed on the slab, loaded in a syringe, and then flooded into the post space to ensure complete coverage and accurate replication of the post shape. The J-shaped wire, coated with a light body, was inserted into the canal, ensuring anticlockwise rotation to completely coat the post space with impression material. This movement facilitates the even distribution of the material, ensuring uniform coverage over all the walls of the post space. Subsequently, the light body was poured around the prepared ferrule to enhance the impression. The heavy body was loaded into stock trays to ensure a detailed and precise impression of the mandibular arch, effectively capturing its intricacies. Similarly, the maxillary impression was also recorded (Figures 6D-6E).

**Figure 7:**
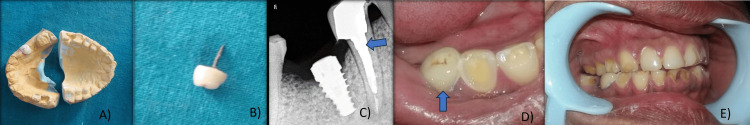
Monoblock post fused crown fabrication and cementation A) Cast made; B) Monoblock cast post and crown; C) Radiograph of post and crown after luting (blue arrow); D) Cementation of monoblock post and crown (blue arrow); E) final occlusion.

The indirect wax pattern was fabricated from the die stone cast using J-shaped wire and blue inlay wax to enhance precision and detail. Subsequently, the monoblock post and crown were cast in metal, tried into the post-space of the tooth, and forwarded for ceramic layering over the crown portion (Figures 7A-7B).

Cementation

After a day, the monoblock cast post-fused crown was cemented using resin-based cement, selected for its durability and adhesive properties (Figure 7C). Thoroughly drying the post-space using air pressure from a three-way syringe is crucial for ensuring optimal bonding and the longevity of the restoration. This process not only eliminates moisture but also clears any remaining debris on the walls, ensuring a pristine surface for subsequent procedures and fostering better adhesion and restoration longevity. A cotton pellet wrapped around a 30K file was rotated in the canal to absorb any remaining moisture and promote a dry environment for the subsequent steps.

Resin cement (3M ESPE Rely X Ultima Clicker) was mixed on a mixing pad, ensuring strict adherence to the manufacturer’s instructions to maintain the desired properties and performance of the cement. It was then applied to the post space using a J-shaped wire in an anticlockwise motion, ensuring even distribution along the walls. Application of resin cement to the post before fitting it into the post space facilitated better flow and seating, ensuring maximum contact and minimal voids. This technique promotes cement flow from the post tip to the crown, reducing voids between the luting surfaces of the post and tooth walls. Excess cement was carefully removed from the cervical margins using dental floss and a carver to ensure a proper fit and prevent any potential gingival issues. High points were traced and corrected (Figure 7D-7E). 

Post-operatively, it is essential to use the night guard during sleep to alleviate pressure on the post-endodontic restoration, thereby mitigating further dentine erosion from gastric acid reflux. Additionally, the patient was counseled against using abrasive toothpowder in the future to prevent additional enamel damage, which could exacerbate erosion and compromise oral health. To alleviate dentinal hypersensitivity, a remineralizing and desensitizing toothpaste was prescribed to fortify the enamel by replenishing minerals and diminishing sensitivity, thereby enhancing overall oral comfort.

## Discussion

The monoblock cast post-fused crown was chosen in this case instead of metal or all ceramic crowns because short preparations are more prone to crown dislodgement [1]. In this case, monoblock cast post-crown was chosen over surgical crown lengthening. So, crown lengthening should be chosen wisely, depending on the remaining tooth structure and occlusal clearance [5], as, gingival inflammation, pocket formation, and plaque accumulation may occur due to the subgingival margin of the crown [5].

Monoblock post-fused crowns were selected instead of all-metal crowns near implants to mitigate galvanization in the oral cavity, which may result from exposure to the salivary atmosphere [13]. Galvanic currents in such situations can lead to periimplantitis, erosion of adjacent teeth, soft tissue lesions, and even the failure of neighboring implants [13].

A separate crown after pre-fabricated and cast post-placement would need extra unaffordable occlusal clearance in this case. Casting a post and crown coping as a single unit is recommended in cases of mutilated teeth with limited vertical space between their upper or lower occluding counterparts [6]. Monoblock post and crown systems are a conservative approach to tooth structure, requiring no extra crown preparation post-cementation [2]. They unify the post and crown, reducing interfaces and increasing stability, reducing post and core separation [9], and minimizing secondary caries [6], with the ferrule effect acting as anti-rotational [4,6]. The monoblock cast post-fused crown for premolars represents a distinctive ceramic-layered crown, differing from Richmond crowns [8], which are typically metal or ceramic-faced. This crown offers complete ceramic coverage on the chewing surface [9]. Moreover, the ceramic layering over the core plays a crucial role in preventing galvanic issues by isolating the metal components, thereby ensuring patient comfort and long-term oral health [13,14].

This design harnesses retention through the ferrule effect [2] and incorporates anti-rotational movement during impression-making [7], effectively reducing the risk of cement failure between the core and crown [6]. Additional benefits of monoblock cast post-fused crowns include a custom-made root configuration, reduced cervical stress and deformation of the post, and ample space for ceramic firing over the coronal portion [6,16]. Despite being time-consuming and costly, this technique offers superior aesthetics compared to metal crowns, particularly in cases with limited incisal clearance [6].

The limitations involved are: These post-endo restorations possess a modulus of elasticity 10 times greater than that of natural dentin. Excessive stress during chewing can lead to a wedging effect, potentially resulting in ceramic fractures or damage to tooth roots. Therefore, it is crucial to utilize monoblock posts and crowns judiciously, only in cases where they are specifically indicated, to achieve optimal results. Furthermore, retrieval of these restorations can prove challenging [7]. 

Studies suggest implants can influence the decay of adjacent teeth [10], and the patient in this case has been avoiding brushing and eating from the implant side for several days. Due to the loss of proximal contacts and the prolonged absence of an adjacent crown, dental caries may occur and progress to pulpitis due to food accumulation and biofilms of plaque [10].

In this case, the erosion resulting from gastric reflux was seen on the buccal, labial, and occlusal surfaces of the teeth, but not on the palatal or lingual surfaces. This unique and rare finding can be attributed to a study by Cheung (2005), which suggested that plaque accumulation on lingual and palatal surfaces might protect against GERD erosion in these areas [12]. Additionally, GERD patients may exhibit poor oral health due to plaque and calculus accumulation and acid corrosion of the metal crowns [12, 14]. In this case, GERD and generalized erosion and wear in the vertical dimensions of the dentition have a significant impact, potentially resulting in issues such as poor oral hygiene and dentinal hypersensitivity [11]. While dental procedures are in the supine position, acid reflux can bother the patient, so the headrest of the dental chair is elevated [11,14]. This position prevents the regurgitation of gastric acid in the mouth [14]. Night guards protect the dentition from extra-occlusal forces and GERD, which can cause corrosion of metal crowns, underscoring the direct impact of the condition on dental materials [11,14]. Digital radiographs from cast post and core systems can serve as unique identification marks for dentition in forensic applications [17].

## Conclusions

A “monoblock cast post-fused porcelain crown,” a specialized technique, is designed to address the unique challenges faced by GERD patients in dental restoration, promoting not only oral health but also overall well-being. The monoblock cast post-fused crown not only offers superior aesthetics but also represents a highly suitable choice for post-endodontic restorations, especially in cases with limited occlusal clarity and weakened teeth. This approach eliminates the potential for galvanic currents from neighboring implants, ensuring superior outcomes, fortifying structural integrity, and preserving the natural aesthetic of the restoration. This less painful and bloodless technique offers a more natural restoration option.
